# The Usefulness of the Short Form-8 for Chronic Pain in the Orofacial Region: A Prospective Cohort Study

**DOI:** 10.7759/cureus.45586

**Published:** 2023-09-20

**Authors:** Aiji Sato-Boku, Tatsuya Tokura, Hiroyuki Kimura, Mikiko Ito, Shinichi Kishi, Takashi Tonoike, Norio Ozaki, Yumi Nakano, Hiroshi Hosijima, Naoko Tachi

**Affiliations:** 1 Department of Anesthesiology, Aichi Gakuin University, Nagoya, JPN; 2 Department of Psychiatry, Nagoya University Graduate School of Medicine, Nagoya, JPN; 3 Department of Oral and Maxillofacial Surgery, Aichi Gakuin University, Nagoya, JPN; 4 Department of Psychological and Physical Sciences, Aichi Gakuin University, Nagoya, JPN; 5 Institute for Glyco-core Research (iGcORE), Nagoya University Graduate School of Medicine, Nagoya, JPN; 6 Department of Psychology and Human Relations, Nanzan University, Nagoya, JPN; 7 Department of Dento-oral Anesthesiology, Tohoku University Graduate School of Dentistry, Sendai, JPN

**Keywords:** short form-8, quality of life, chronic pain, persistent idiopathic facial pain, burning mouth syndrome

## Abstract

Background and purpose

Given that chronic pain has become a major problem in recent years, affecting approximately 30% of the general population, this study used the Japanese version of the Short Form-8 (SF-8) to investigate (1) the quality of life (QOL) of patients with burning mouth syndrome (BMS) or persistent idiopathic facial pain (PIFP) (compared with a Japanese control group) and (2) whether therapeutic intervention improves the QOL and reduces pain (comparison between 0 and 12 weeks) of patients with BMS or PIFP.

Materials and methods

A total of 63 patients diagnosed with either BMS (n=45) or PIFP (n=18) were included in this study. The diagnostic criteria for BMS and PIFP were established based on the third edition of the International Classification of Headache Disorders.

Results

Our study results showed that while Physical Component Summary (PCS) in patients with BMS or PIFP improved with treatment, it did not improve to the national standard value (NSV) after 12 weeks of intervention. In contrast, the Mental Component Summary (MCS) improved to the same level as the NSV after 12 weeks of intervention.

Conclusions

We found that therapeutic intervention improves MCS and reduces pain; however, improving PCS requires time.

## Introduction

Chronic pain has become a major problem in recent years, affecting approximately 30% of the general population [1]. The majority of these patients are difficult to treat, and even when they can be treated, 50% of them experience only partial improvement and reduced quality of life (QOL) [2,3]. However, understanding chronic pain is difficult; thus, it is underdiagnosed and under-treated because pain cannot be commensurated with organic abnormality [4,5]. Chronic pain can be a comorbidity of mental illness such as depression and can also affect various aspects of patients’ daily lifestyles [6].

Chronic pain in the orofacial region includes various conditions such as burning mouth syndrome (BMS), persistent idiopathic facial pain (PIFP), and nonorganic temporomandibular joint disorder. BMS and PIFP are commonly encountered in daily clinical practice [7]. Since these patients often complain of physical symptoms only, which are the main complaints, establishing an accurate diagnosis, identifying the treatment, and evaluating the degree of improvement are difficult even after the treatment intervention.

The concept of health-related quality of life (HRQOL) has been generally used as a multidimensional assessment of how disease and treatment affect a patient’s sense of overall function and well-being [8]. It is also an inclusive concept based on the patient’s subjective judgment. In other words, HRQOL quantifies the impact of an illness on the performance of activities of daily living. The Short Form-8 (SF-8) is a comprehensive, versatile, and practical tool globally used for measuring HRQOL [9], allowing the comparison of normative values from large national surveys with results from more focused outcome studies. The SF-8 is based on the SF-36, a 36-item version of the rating scale, but is more convenient because it provides results equivalent to those of the SF-36. Although the authors provided treatment interventions for many patients with chronic pain in the orofacial region, pre- and posttreatment evaluations were based on conventional clinical diagnostic assessments performed by physicians and dentists and were inadequate from the patient’s viewpoint. In addition to pain reduction, the goal of treatment is to improve QOL.

In this study, the SF-8 Japanese version [10] was used to investigate (1) the QOL of patients with BMS or PIFP compared with a Japanese control group and (2) whether the therapeutic intervention improves the QOL and reduces pain (comparison between 0 and 12 weeks).

This article was previously posted to the medRxiv preprint server on May 22, 2023.

## Materials and methods

Ethical guidelines

This study was conducted as per the principles of the Declaration of Helsinki and was approved by the Ethical Review Committee of Nagoya University Graduate School of Medicine (no. 234, 234-2, 2004-0234-2, and 2004-0234-3) and the Ethical Review Committee of the School of Dentistry, Aichi Gakuin University (no. 41). All the participants provided written informed consent.

Study design and patients

Patients who had a first visit to the liaison outpatient clinic of the Department of Oral and Maxillofacial Surgery, Aichi Gakuin University Dental Hospital, between May 10, 2010, and April 21, 2021, were diagnosed with BMS or PIFP by a dentist using the third edition of the International Classification of Headache Disorders (Table 1) [11], were diagnosed with somatic symptom syndrome with predominant pain by a psychiatrist using the Diagnostic and Statistical Manual of Mental Disorders, Fifth Edition (DSM-5) [12], and whose consent for the study was obtained were included in the study.

**Table 1:** Diagnostic criteria of BMS and PIFP based on ICHD-3 BMS: burning mouth syndrome, ICHD-3: The International Classification of Headache Disorders third edition, PIFP: persistent idiopathic facial pain

BMS				PIFP			
								A.	Oral pain fulfilling criteria B and C			A.	Facial and/or oral pain fulfilling criteria B and C		
B.	Recurring daily for two hours per day for >3 months			B.	Recurring daily for two hours per day for >3 months										
								C.	Pain has both of the following characteristics: 1. burning quality and 2. felt superficially in the oral mucosa			C.	Pain has both of the following characteristics: 1. poorly localized and not following the distribution of the peripheral nerve and 2. dull, aching, or nagging quality		
D.	Oral mucosa is of normal appearance and clinical examination including sensory testing is normal			D.	Clinical neurological examination is normal										
								E.	Not better accounted for by another ICHD-3 diagnosis			E.	A dental cause has been excluded by appropriate investigations		
				F.	Not better accounted for by another ICHD-3 diagnosis										

Patients with a history of psychotic disorders or cognitive dysfunction were excluded from this study. For patients who visited our outpatient clinic before 2013, the Diagnostic and Statistical Manual of Mental Disorders, Fourth Edition, Text Revision (DSM-IV-TR) [13] was used instead of the DSM-5. These psychiatric diagnoses were re-categorized based on the DSM-5 by confirming each patient’s clinical record. During the study period, 63 patients (45 with BMS and 18 with PIFP patients) participated in the study.

A structured clinical interview was conducted to establish a psychiatric diagnosis based on DSM-5. Regarding therapeutic interventions, serotonin noradrenaline reuptake inhibitors (SNRIs) are more commonly used than tricyclic antidepressants because of their fewer adverse effects, although antidepressants have been widely used for the treatment of chronic pain. Our previous study reported that SNRI duloxetine is effective for chronic pain in the orofacial region [14-17]. Duloxetine was also used in the present study and administered to patients with the above diagnosis from the initial visit (0 weeks). The initial dose of duloxetine was 20 mg, once daily. The dose was increased to a maximum of 40 mg once daily and symptoms and adverse effects were observed.

Outcome measures

As the main outcome, for patients who received the therapeutic intervention, the SF-8 at the initial visit and after 12 weeks of intervention was evaluated and compared with those of the Japanese control participants. The SF-8 consists of the following items: general health, physical function, daily role function (physical), physical pain, vitality, social function, mental health, and daily role function (mental) (Table 2) [9].

**Table 2:** Component concepts and questions of SF-8 The score for each item is indicated by a deviation score based on the national standard value (50). In addition, a summary score can be calculated by multiplying the score of each item by a coefficient summary score. The items closely related to the physical summary score are 1 to 4, and the items closely related to the mental summary score are 5 to 8 SF-8: the Short Form-8

Component concepts				Questions						
											1	General health			Overall, how has your health been over the past week?						
2	Physical function			In the past month, how often have you been prevented from doing any of the daily activities that involve physical exertion (e.g., walking, climbing stairs) for physical reasons?																	
											3	Daily role function (physical)			In the past month, how often have you been prevented from doing your usual work (including household chores) for physical reasons?						
4	Physical pain			In the past month, how much physical pain have you had?																	
											5	Vitality			In the past month, how well have you been?						
6	Social function			In the past month, how often have your usual social interaction with family and friends been prevented by physical or psychological reasons?																	
											7	Mental health			In the past month, how often have you been suffering from psychological problems (feeling anxious, depressed, irritable)?						
8	Daily role function (mental)			In the past month, how often have your daily activities (usual activities such as work, school, household chores, etc.) been prevented by psychological reasons?																	

These eight items were scored using norm-based scoring (NBS; scoring based on the national standard value (NSV) 50) for each item. Based on the eight items, two summary scores, “Physical Component Summary (PCS)” and “Mental Component Summary (MCS),” were calculated to indicate physical and mental health, respectively. Higher scores indicated higher QOL for both PCS and MCS. A group of healthy participants matched in number with the patient group was also created from the NBS data and used as the NSV.

Additionally, pain intensity was evaluated using a visual analog scale (VAS). To evaluate depression in 63 patients with BMS and PIFP, Beck’s depression inventory (BDI; cutoff value, ≤10) was used as a subjective index, and the Hamilton Depression Rating Scale (HDRS; cutoff value, ≤7), which uses semi-structured interviews by a trained psychiatrist, was used as a highly precise objective index [18,19].

The score for each item of the SF-8 was indicated by a deviation score based on the NSV (50). In addition, a summary score was calculated by multiplying the score of each item by the coefficient summary score. Items closely related to the physical summary score were 1-4, and items closely related to the mental summary score were 5-8.

Statistical analysis

Data are expressed as median (IQR) or number. Because the sample size was small and the data did not follow a normal distribution, we adopted the median value instead of the average value. For statistical testing, the Mann-Whitney U test was used to compare the differences between the two independent groups for continuous variables. The two-sided statistical significance level was set at p ≤ 0.05. Statistical analysis of the recorded data was performed using SPSS Statistics version 26 (IBM Corp. Released 2019. IBM SPSS Statistics for Windows, Version 26.0. Armonk, NY: IBM Corp.).

## Results

Table 3 shows the patients’ demographic characteristics and psychiatric diagnoses.

**Table 3:** Characteristics of the patients in this study BMS: burning mouth syndrome, IQR: interquartile range, PIFP: persistent idiopathic facial pain

	BMS		PIFP		
Patient's demographics	Median	IQR	Median	IQR	p-value
Age (year)	64	54-70	57.5	51.3-64.8	p<0.01
Gender	Number	%	Number	%	p-value
Male	6	14	1	6	p=0.37
Female	39	86	17	94	
Psychiatric diagnosis	Number	%	Number	%	p-value
Major depressive disorder	1	2	0	0	p=0.44
Somatic symptom disorder with predominant pain	35	77	17	95	
Somatic symptom disorder (other than those above)	2	5	0	0	
Major depressive disorder + somatic symptom disorder with predominant pain	7	16	1	5	

**Figure 1:**
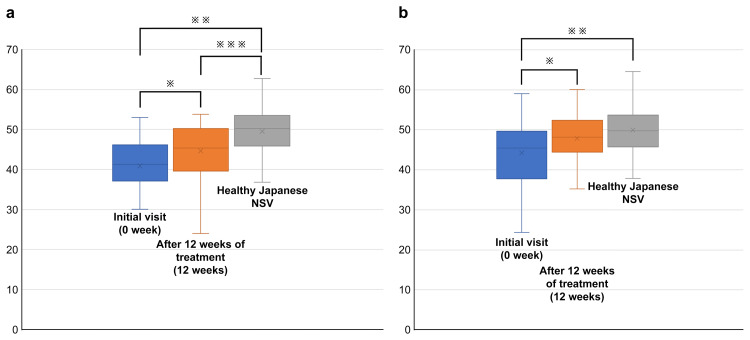
(a) Comparison of SF-8 PCS scores between the initial visit, after 12 weeks of treatment, and healthy Japanese data. (b) Comparison of SF-8 MCS scores between the initial visit, after 12 weeks of treatment, and healthy Japanese data a: ※P < 0.01 (0 vs. 12 weeks), ※※P < 0.01 (0 week vs. NSV), ※※※P < 0.01 (12 weeks vs. NSV) b: ※P = 0.01 (0 vs. 12 weeks) ※※P < 0.01 (0 week vs. NSV) NSV: national standard value

As shown in Figure 1, the SF-8 PCS was 41.3 (37.1-45.9) at 0 weeks and 45.3 (40.2-50.1) at 12 weeks, and the NSV was 50.28 (45.86-53.28).

Statistically significant differences were observed between 0 and 12 weeks, between 0 weeks and NSV, and between 12 weeks and NSV. The SF-8 MCS was 45.4 (38.4-49.6) at 0 weeks and 48.1 (44.4-52.3) at 12 weeks, and the NSV was 49.86 (45.96-53.49). Statistically significant differences were observed between 0 and 12 weeks and between 0 weeks and NSV. However, no statistically significant difference was observed between 12 weeks and the NSV group.

**Figure 2:**
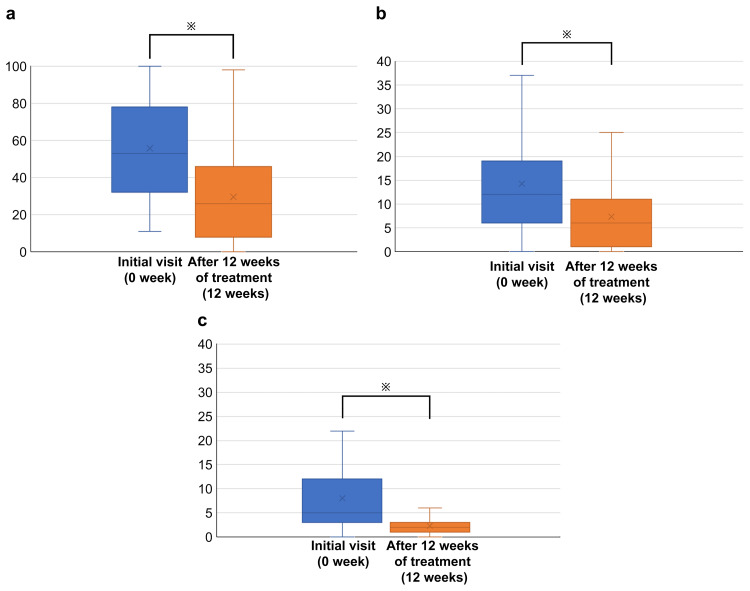
(a) Comparison of VAS scores at the initial visit and after 12 weeks of treatment. (b) Comparison of BDI scores between the initial visit and after 12 weeks of treatment. (c) Comparison of the HDRS scores between the initial visit and after 12 weeks of treatment a: ※P < 0.01 (0 vs. 12 weeks) b: ※P < 0.01 (0 vs. 12 weeks) c: ※P < 0.01 (0 vs. 12 weeks)

Figure 2 shows the VAS, BDI, and HDRS scores between the initial visit and after 12 weeks of treatment.

The VAS score was 53 (32.5-77) at 0 weeks and 26 (8.5-46) at 12 weeks. The BDI was 12 (6.5-18.5) at 0 weeks and 6 (1.5-10.5) at 12 weeks. The HDRS scores were 6 (3-12) at 0 weeks and 2 (1-3) at 12 weeks. All the parameters showed statistically significant differences between 0 and 12 weeks.

## Discussion

This study showed that although PCS in patients with BMS or PIFP improved with treatment, it did not improve to NSV after 12 weeks of intervention, whereas MCS improved to the same level as NSV after 12 weeks of intervention. The results demonstrated that the treatment intervention improved the BDI and HDRS scores, a measure of depression. Emotional factors have been reported to be strongly involved in the deposition and relief of chronic pain and may have diverse effects on pain expression [20]. This study indicates that when no organic cause has been identified for physical symptoms, prompt collaboration between psychosomatic medicine and psychiatry is extremely important, rather than making a definitive diagnosis and treatment by dentistry alone.

Previous reports using the SF-8 have included evaluations for rheumatism [21] and stroke [22], and the study results indicated that it can be used adequately in the field of dentistry. Visual assessment methods, such as the VAS [23] and the face scale [24], have been previously used to assess patients’ mental satisfaction. To evaluate medical interventions, subjective factors including psychosocial aspects should be measured. A scale such as SF-8 can be a good means of communication between physicians and patients.

MCS recovered, while PCS did not recover to NSV in this study. Thus, emotional turbulence may cause muscle hyperactivity induced by the central nervous system, resulting in parafunctional habits [25]. Depression is also reported to be a more likely consequence than a precursor of living with pain; therefore, the mind needs to gain supremacy over the body to compensate for pain. In other words, the emotional side of pain should be managed first, rather than both the mental and physical sides, and our study may have been the result of an intervention from the mental side. However, restoring PCS scores is still necessary to improve patients’ QOL. This may be because many patients do not yet achieve the level at which treatment can be terminated, although symptoms tend to decrease after up to 12 weeks of treatment, and treatment for PCS may take a longer period to recover to a level comparable to NSV. One report revealed that it took two years for PCS to finally approach the national norm in a patient with postoperative head and neck cancer [26].

This study has several limitations. First, the number of patients and the study duration were limited (as noted above, PCS may improve with longer follow-up), and it was a single-center study. Second, it was not possible to properly evaluate the effect of treatment with duloxetine because this study was not an intervention study such as a randomized controlled trial. In addition, as the SF-8 is a scale that can be used for a wide range of patients, from normal participants to patients with chronic diseases, it is not disease-specific for chronic pain in the orofacial region.

## Conclusions

In conclusion, we used the SF-8 Japanese version to investigate (1) the QOL of patients with BMS or PIFP compared with a Japanese control group and (2) whether the therapeutic intervention improves QOL and pain reduction (comparison between 0 and 12 weeks). The results showed that statistically significant differences were observed between the QOL of patients with BMS or PIFP and a Japanese control group. Therapeutic intervention improved MCS and reduced pain; however, the PCS improvement took time.
